# King George's Fund for Sailors

**Published:** 1920-03-20

**Authors:** 


### King George's Fund for Sailors.

A meeting of the General Council of King George's Fund for Sailors was held in the Court Room of Trinity House on March 10. In the absence of His Royal Highness the Duke of Connaught, Captain A. W. Clarke, the Deputy-Chairman, presided. The principal object of the meeting was to adopt the report for the year 1919, in which the General Council recorded the fact that widespread and generous support continued to be accorded to the Fund, both from within the United Kingdom and throughout the Empire. The total amount received during the year was £258,269. Donations received during the year included £65,000 from the people of Canada, £26,500 from the people of Bengal, £22,000 from the people of Queensland, £11,000 from the Colony and Protectorate of Nigeria, £10,684 from the Dominion of New Zealand, £6,400 from the Crown Colony of Jamaica, £5,000 from the British Patriotic Committee, Buenos Aires, and £2,500 from the Guild of Loyal Women in South Africa. The cost of administration and collection of the Fund worked out at an average of less than 1 per cent., or 2d. in every £1. The total amount distributed has been £160,000, including the grants of £49,940 for the year 1919. In the later allocations were included a grant of £5,000 to the Henry Radcliffe Convalescent Home at Limpsfield, Surrey; £2,500 to the Seamen's Hospital Society, towards the establishment of a sanatorium for seamen suffering from consumption; £2,500 towards the establishment of the King George's Merchant Seamen's Hospital at Malta; £2,000 to the Committee of the Marseilles Hospital; £1,000 to the Port Said Hospital towards the purchase of a new site for further extensions; £500 towards the extension of the Alexandra Maternity and Children's Homes; £1,000 to widows of merchant seamen through the Pension Fund administered by the Royal Alfred Institution; £1,000 to the Samaritan Fund of the same Society; £1,000 to the Grand Fleet Emergency Relief Fund for relief in connection with the men of the Royal Navy and their dependants; and £200 to the Destitute Fund for merchant seamen in distress of the Sailors' Home and Red Ensign Club. The General Council has sanctioned the sum of £10,000 to be set aside to meet applications of urgency that may be received in 1920. In proposing the adoption of the report, the Deputy-Chairman drew attention to the fact that although many war funds had terminated their activities during the past year, King George's Fund for Sailors was a permanent fund, being incorporated under Royal Charter, and it was hoped to continue its activities in perpetuity.

